# Developing and Validating the Scale of Organizational Support Needs for Nurses Returning to Work After Childbirth

**DOI:** 10.1155/jonm/5842526

**Published:** 2025-08-18

**Authors:** Xinli Wan, Jiayi Yang, Yingli Pan

**Affiliations:** ^1^Cardiothoracic Surgery Ward, Yichang Central People's Hospital, Yichang, Hubei, China; ^2^Thoracic Surgery Ward, The First Hospital of China Medical University, Shenyang, Liaoning, China; ^3^Nursing Department, Fourth Affiliated Hospital of China Medical University, Shenyang, Liaoning, China

**Keywords:** instrument development, nurses, psychometric validation, return to work

## Abstract

**Aims:** To describe the development and testing of the psychometric properties of the scale of organizational support needs for nurses returning to work after childbirth.

**Design:** Instrument development and validation.

**Methods:** The study was divided into three steps: the first step involved developing items using qualitative descriptive research and literature review to form an item pool of the scale; the second step entailed modifying the items through Delphi expert consultation, content validity, and a presurvey to create the draft of the scale; and the third step was to adjust the dimensions through exploratory factor analysis and confirmation factor analysis to form the final scale and evaluate its psychometric properties. Data were collected from August 2022 to May 2025.

**Results:** The scale consisted of 14 items across three dimensions: manager support, work condition support, and colleague support. The content validity index at the item level ranged from 0.83 to 1.00, and the average content validity index at the scale level was 0.92. Three factors were revealed using the exploratory factor analysis, which accounted for 72.912% of the variance. Cronbach's alpha was 0.939 for the total scale and 0.871 to 0.920 for the three dimensions. The Spearman–Brown coefficient was 0.908 for the total scale and 0.872 to 0.922 for the three dimensions. The structural equation modeling showed a good model fit, confirming construct validity (*χ*^2^/df = 1.0673, RMSEA = 0.0180, SRMR = 0.0351, CFI = 0.9969, TLI = 0.9962). The scale developed in this study is valid and reliable enough to be used widely.

**Conclusions:** The scale of organizational support needs for nurses returning to work after childbirth demonstrates good psychometric properties and can be used to assess the organizational support needs of nurses returning to work.

## 1. Introduction

Women make up nearly 70% of the global health and social workforce as well as a similarly large proportion of nursing and midwifery occupations [1]. By the end of 2020, there were 5.02 million registered nurses in China [2], of whom 97.1% were women, and 85.5% were of childbearing age (18–45 years) [3]. In response to an increasingly aging society, the Chinese government is encouraging childbearing and improving the country's demographic structure. On May 31, 2021, China officially implemented a three-child policy, allowing couples to have three children. The number of births in the nursing population is bound to rise; consequently, the number of nurses returning to work after childbirth will gradually increase. For women returning to work after childbirth, China offers protective policies, such as 1 hour of breastfeeding during working hours and not being assigned night shifts for 1 year after childbirth. In the experience of nurses returning to work after childbirth, support from government policies alone was insufficient for nurses to return successfully to work and breastfeeding after childbirth [4, 5]. Therefore, this study focused on the population of nurses returning to work after childbirth in China.

The issue of women returning to work after childbirth has been a popular research topic in many countries. Working women usually breastfeed after returning to work, and improving breastfeeding rates in infants has been the focus of global research [6]. Rollins's study found that returning to work was an obstacle to breastfeeding [7]. However, combining breastfeeding and returning to work can be challenging for working women [8], requiring not only workplace support for breastfeeding [9] but also support from colleagues and managers [10]. After a long absence from the workplace due to childbirth, working women face an adjustment period upon returning to work, which not only involves adapting to their new social role but also involves readjusting to their jobs [11]. The adjustment period requires not only the efforts of working women themselves but also the support of the organization. Currently, the management of women who return to work after childbirth is often informal [12]. The management of human resources concerning pregnant and lactating nurses presents a significant challenge for nursing managers. It has been observed that nurses who gave birth to their second or third child do not benefit from the same management policies and deployment as those who gave birth to their first child [4]. Nurses who gave birth to their second child were scheduled for night shifts just 1 month after returning to work, despite the protocol that requires them to wait until their child is 1 year old [4]. Nurses who gave birth to their first child were allowed to work in an auxiliary position for 6 months after returning to work; however, nurses who gave birth to a second child had to care for patients directly upon returning [4]. The practical experiences and specific needs of nurses reentering work after childbirth must be highlighted to their colleagues and managers, and these nurses should receive enhanced social support [5].

Organizational support theory (OST) [13] is a theory that explains employer–employee relationships based on social exchange, with a central structure of perceived organizational support [14]. This theory assumes that, based on the principle of reciprocity, employees who perceive organizational support feel obligated to reciprocate toward the organization. Perceived organizational support mainly comes from four aspects: organizational justice, organizational rewards and working conditions, supportive work–life practices, and manager support [15].

Therefore, this study focused on the organizational support needs of nurses returning to work after childbirth, using OST as a theoretical basis to develop a scale and evaluate its psychometric properties. The scale could be a reliable measurement tool for investigating the organizational support needs of nurses returning to work after childbirth in China, provide a basis for managers to make management decisions, and lay the foundation for the construction of a management program for nurses returning to work after childbirth.

## 2. Materials and Methods

### 2.1. Study Design and Setting

Using Devellis' scale development methodology, this study aimed to develop an organizational support needs scale for nurses returning to work after childbirth [16]. From November 2021 to March 2022, items were extracted using descriptive qualitative research and literature review to construct the item pool. From April 2022 to June 2022, the items in the pool were screened and revised using a modified Delphi expert consultation to form a draft of the scale. From August 2022 to May 2025, the draft of the scale was subjected to measurement of psychometric properties, and modifications were made to finalize the organizational support needs scale for nurses returning to work after childbirth.

### 2.2. Participants

The inclusion criteria for participants were as follows: (1) registered nurses; (2) those returning to work within 6 months or less following childbirth; (3) individuals who have given birth to a healthy newborn; and (4) provision of informed consent.

The exclusion criteria included (1) nurses who are on leave due to personal illness and (2) nurses currently undergoing training.

### 2.3. Item Development

Project development was accomplished through a combination of qualitative descriptive research and literature review. A purposive sample of 15 nurses was selected for face-to-face, semistructured interviews, each lasting between 30 and 50 min. The interview data were collected between November 2021 and March 2022. After reviewing a great deal of relevant qualitative research literature, an outline of the interview was developed. The outline of the interview was as follows: How was your work experience when you returned to work after childbirth? What difficulties did you encounter while returning to work after childbirth? How did you solve them? What help would you like the organization to provide for you? Do you have anything to address these issues? Before the start of the formal interviews, two interviews were conducted as a preexperiment to gradually modify the specific questions based on the participants' responses and confirm the feasibility of the interview outline. The recordings were transcribed verbatim by the researcher within 24 h of the end of each interview. Items were extracted using the conventional content analysis method in which the researcher immersed themselves in the text to extract items relevant to the study without preconceived categories [17]. The data were analyzed using content analysis, and 31 items and five categories were extracted: (a) manager support, (b) work condition support, (c) work environment support, (d) colleague support, and (e) emotional support.

To supplement these items, the literature relevant to this study was reviewed by systematically searching available literature databases. Seven Chinese and English databases were searched from database construction until March 2022, including Web of Science, PubMed, Cumulative Index to Nursing and Allied Health Literature, China National Knowledge Infrastructure, Wanfang, China Science and Technology Journal Database, and SinoMed. The researchers searched the literature using the keywords “return to work,” “nurses,” and “demand.” Literature screening and item extraction were independently performed by two trained researchers, followed by cross-checking. In case of disagreement, group discussions or third-party judgments were conducted. Literature inclusion criteria included the following: (1) the study participants were nurses who returned to work after childbirth, gave birth to healthy newborns, and did not have any disease; (2) the study covered the work experience of nurses who returned to work after childbirth and the need for organizational support; (3) there was no restriction on the type of literature; and (4) the language of the literature was limited to Chinese or English. Literature exclusion criteria were as follows: (1) literature that is not available in full text or with incomplete data and (2) repeatedly published literature. The specific steps of literature screening were as follows: (1) import 1398 retrieved articles into EndNote 20 literature management software to exclude 247 duplicates; (2) according to the inclusion and exclusion criteria, read the titles and abstracts to exclude 1056 irrelevant articles; and (3) read the full text for rescreening to exclude 20 articles whose research participants were inconsistent with this study and 62 articles whose content was not consistent with this study. Finally, 13 articles were obtained [18–30]. The researcher carefully studied these articles and extracted 11 items related to the organizational support needs of nurses returning to work after childbirth. Nine of these 11 items were duplicates of the nine items extracted from the qualitative descriptive research. Therefore, after the literature review, 2 different items were extracted.

The item pool consisted of five dimensions (manager support, work condition support, work environment support, colleague support, and emotional support) and 33 items.

### 2.4. Delphi Expert Consultation

In this study, a modified Delphi method was used to revise the draft of the scale [31]. The corresponding questionnaire consisted of three parts: a letter to an expert, a consultation form for the draft of the scale, and an expert information questionnaire. The questionnaire was formally distributed online from April to June 2022. After the first round of consultation, the researcher summarized the experts' opinions, and the research group discussed and modified the questionnaire according to the results of the first round of consultation and the experts' opinions to form the second round of consultation questionnaire. The second round of questionnaires was distributed 2-3 weeks after the first round, and the results of the first round were sent back to the experts as a reference. After the second round of expert consultation, the experts' opinions were summarized, and the distribution of questionnaires was stopped when the experts' opinions converged [32]. Fifteen experts working in the field of nursing management or clinical nursing were recruited from nine provinces and cities in China. The consultations were conducted between April and June 2022. All experts had an average of thirty-one years of experience: nine (60.00%) held senior titles, six (40.00%) held associate senior titles, two (13.33%) had PhDs, four (26.67%) had master's degrees, and nine (60.00%) had bachelor's degrees. The consultation questionnaire used a Likert 5-point scale (5 = very important, 4 = important, 3 = normal, and 2 = not important), in which experts rated the importance of the items and provided recommendations.

The recovery rate of the two rounds of questionnaires was 100.00%, and the authority coefficient of the experts was 0.92, indicating that the experts had high enthusiasm and authority. Based on the results of the expert consultation, two dimensions were merged: one dimension and its seven items were deleted, one item was added, two items were deleted, four items were merged, and one item was split. After the expression of the 11 items was modified, the test version of the scale consisted of three dimensions (manager support, work condition support, and colleague support) and 24 items.

### 2.5. Content Validity Analysis

The content validity index (CVI) is the most widely used method for quantifying scale validity [33]. In March 2025, six experts were invited to rate the correlation between the items and the scale to evaluate its content validity. Experts scored the relevance of each item to the corresponding content dimension on a 4-point rating scale, with 1 being “not relevant” and 4 being “very relevant.” When the number of experts was greater than or equal to six, the CVI at the item level (I-CVI) exceeded 0.78 [34]. The average CVI for the scale (S-CVI/Ave) should be at least 0.90 [35].

Six experts had an average of 26 years of work experience: two (33.33%) held senior titles, four (66.67%) held associate senior titles, and six (100.00%) had bachelor's degrees. The results showed that the CVI for each item level (I-CVI) ranged from 0.83 to 1.00, and the average CVI for the scale level (S-CVI/Ave) was 0.92.

### 2.6. Presurvey

In August 2022, ten nurses returning to work after childbirth from a tertiary care hospital in Shenyang were conveniently selected to conduct a presurvey. The purpose was to modify the formulation of the items according to the nurses' suggestions. A presurvey was conducted using face-to-face questionnaires to obtain the nurses' views. For clarity, the wording of an item was changed from “provide me with child care day care” to “provide me with child care day care (child care services).” These ten questionnaires were part of the psychometric property evaluation.

### 2.7. Psychometric Property Evaluation

From August 2022 to May 2025, convenience sampling was adopted to select nurses who returned to work after childbirth from several third-grade A hospitals in Shenyang and Anshan of Liaoning Province and Yichang of Hubei Province for a questionnaire survey. The instruments used were a general information questionnaire designed by the researcher and a test version of the scale developed for this study. We distributed 435 questionnaires and received 414 valid responses, resulting in a questionnaire validity rate of 95.17%. The 206 questionnaires collected from August 2022 to December 2024 were used for item analysis and exploratory factor analysis (EFA). The 208 questionnaires collected from January to May 2025 were used for confirmatory factor analysis (CFA). To validate the psychometric properties, the sample size was calculated as 120–240, based on it being five to ten times the number of items [36]. The sample size of this study met the requirements.

#### 2.7.1. Item Analysis

Three methods were used to filter and analyze the items: (1) the critical ratio decision value method, (2) the Pearson correlation coefficient analysis to calculate the correlation coefficients between the scores of each item of the scale and the total score, and (3) the calculation of Cronbach's alpha coefficient after the deletion of items. Delete items with critical ratio decision value less than 3.000 or differences that were not statistically significant (*p* > 0.05); delete items with item-total correlation coefficient less than 0.400 or differences that were not statistically significant (*p* > 0.05) [37]. If the Cronbach alpha coefficient was significantly increased after deleting an item and the adjusted item-total correlation was less than 0.300, the item was deleted [38, 39].

#### 2.7.2. Validity Analysis

EFA was used to evaluate the construct validity of the scale, and the dimensions and item attributes were adjusted accordingly. Kaiser–Meyer–Olkin (KMO) and Bartlett's tests of sphericity were performed to determine the suitability of the items for EFA. When the KMO value is greater than 0.60, EFA can proceed [40], and a *p* value of Bartlett's test less than 0.05 indicates a correlation between the items, allowing for EFA [41]. Factors were extracted using principal axis factoring and a scree test [42]. The slope of the scree plot flattened after a break, and the factor to the left of the break was retained [43]. Factor loadings were determined using the oblique rotation to obtain the factor-loading matrix [44]. Factors with eigenvalues > 1 were extracted [45], and the loadings of items on each factor were required to be greater than 0.40 [46]; if they were loaded on more than one factor, the difference in loadings was required to be greater than 0.10 [40].

CFA was implemented based on the structural equation modeling (SEM) framework, and maximum likelihood estimation was used for model parameter estimation. The model goodness of fit was comprehensively assessed by the chi-square degrees of freedom ratio (*χ*^2^/df), root mean square error of approximation (RMSEA), standardized root mean square of residuals (SRMR), comparative fit index (CFI), and Tucker–Lewis index (TLI). The *χ*^2^/df value of less than 5 is preferred. The RMSEA value of less than 0.06 is considered appropriate [47]. The SRMR value should be close to or less than 0.08, and both CFI and TLI values should be greater than 0.95 [48]. Based on the validation factor analysis, the average variance extracted (AVE) and composite reliability (CR) can be further calculated to verify the validity of the scale. The standardized factor loading of each item on the corresponding factor should be greater than or equal to 0.70, indicating that the item explains more than 50% of the variance of the factor [49]. The AVE value should be greater than 0.50, indicating that the latent variable explains more than 50% of the variance of its items [49]. And the CR value should be greater than 0.70, indicating that there is a high degree of internal consistency of the items under the same factor [49].

#### 2.7.3. Reliability Analysis

Internal consistency was used to evaluate the reliability of the scale after EFA.

### 2.8. Scale Scoring Method

The scale was scored on a Likert 5-point scale from 5 (strongly need), 4 (need), 3 (generally need), 2 (not much need), and 1 (no need), all of which were positively scored. The dimension scores were obtained by calculating the mean scores of the entries, which all ranged from 1.00 to 5.00 points, where 1 indicates the lowest intensity of need and 5 indicates the highest intensity of need. To facilitate cross-study comparisons, the scores can be further converted to a 0–100 score by the formula Standardized Score=(Mean Score − 1)/4 × 100, with higher scores indicating a more urgent need for organizational support for that dimension.

### 2.9. Data Analysis

SPSS 26.0, AMOS 26.0, and Microsoft Excel were employed to conduct descriptive data analysis, item analysis, validity analysis, and reliability analysis in the evaluation of psychometric properties.

## 3. Results

### 3.1. Demographic Characteristics

The nurses' ages ranged from 25 to 44 years. Most were Han Chinese, with 56 (13.53%) being ethnic minorities. Their work experience ranged from 2 to 22 years. Among them, 286 (69.08%) had junior titles, 121 (29.23%) had intermediate titles, and 7 (1.69%) had associate senior titles. Educational qualifications included 77 (18.60%) nurses with a college degree, 326 (78.74%) with a bachelor's degree, and 11 (2.66%) with a master's degree. There were 353 (85.27%) nurses who gave birth for the first time and 61 (14.73%) for the second time (Table 1).

### 3.2. Item Analysis Results

After calculating the critical ratio decision value, eight items were found to have statistically insignificant differences (*p* > 0.05). The eight items were: item 3 “I need the manager to grant my request for leave at her/his discretion,” item 5 “I need a good working atmosphere,” item 9 “I need supportive psychological help from the manager (listening, explaining, advising, etc.),” item 10 “I hope the manager supervises my work more,” item 11 “Provide me with child care day care (child care services),” item 13 “Provide me with milk storage equipment,” item 14 “Provide me with a variety of working meals,” and item 24 “I need supportive psychological help from colleagues (listening, explaining, advising, etc.).”

The remaining 16 items, after the deletion of 8 items, continue to item analysis. The results showed that the correlation coefficient between each item and the total score was 0.594–0.782 (*p* < 0.05). Cronbach's alpha of the test version of the scale after the deletion of 8 items was 0.942. Cronbach's alpha coefficient was not significantly increased after deleting any of the items. And the adjusted item-total correlation was 0.591–0.766. No items were removed after calculating the item-total correlation coefficient and Cronbach's alpha (Table 2). Therefore, after the item analysis, 16 items remained.

### 3.3. Validity Analysis Results

The construct validity of the scales was evaluated using EFA. The remaining 16 items were subjected to the first EFA after the item analysis. The KMO value was 0.941, and the results of Bartlett's test of sphericity were statistically significant (*χ*^2^ = 2191.153, *p* < 0.001), indicating that the sample was suitable for EFA. The result of the first EFA was that three factors were extracted with a cumulative variance of 68.901%, but the loadings on each factor for item 19 were less than 0.40; therefore, item 19 was deleted for the second EFA.

The second EFA was performed on the remaining 15 items. The KMO value was 0.941, and the results of Bartlett's test of sphericity were statistically significant (*χ*^2^ = 2077.894, *p* < 0.001), indicating that the sample was suitable for a second EFA. The results showed that three factors were extracted with a cumulative variance contribution of 70.723%, but the loadings on each factor for item 18 were less than 0.40; therefore, item 18 was deleted for the third EFA.

**Figure 1:**
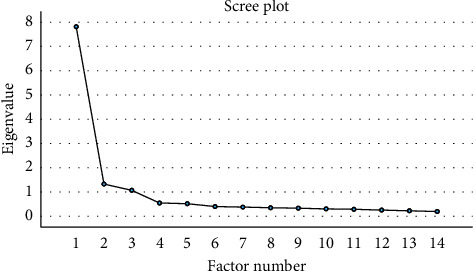
Scree plot.

The third EFA was performed on the remaining 14 items. The KMO value was 0.942, and the results of Bartlett's test of sphericity were statistically significant (*χ*^2^ = 1959.424, *p* < 0.001), indicating that the sample was suitable for a third EFA. The results showed that three factors were extracted with a cumulative variance contribution of 72.912%, and the scree plot (Figure 1) showed that the slope flattened after the third factor, indicating that the scale still had a three-factor structure (Table 3).

**Figure 2:**
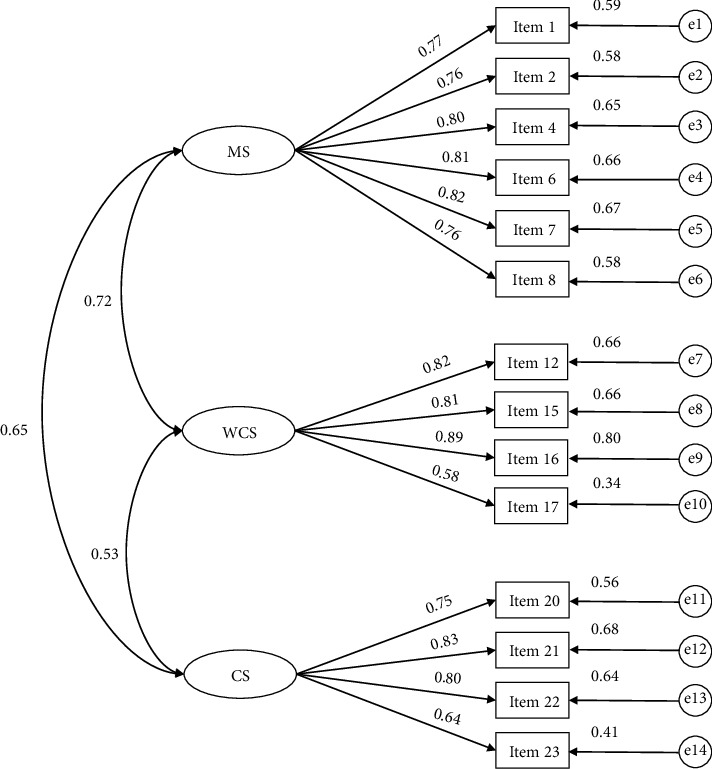
The CFA model of the scale of organizational support needs for nurses returning to work after childbirth. Note: MS, manager support; WCS, work condition support; CS, colleague support.

The remaining 14-item three-factor model was submitted to a CFA. The *χ*^2^/df value was 1.0673, which was less than 5. The RMSEA value was 0.0180, which was less than 0.06. The SRMR value was 0.0351, which was less than 0.08. The CFI value was 0.9969, and the TLI value was 0.9962, both of which were greater than 0.95. Overall, the model had a good fit. The results of the convergent validity are shown in Table 4, and the CFA model is shown in Figure 2. All standardized factor loadings were greater than 0.50, CR values were greater than 0.60, and AVE values were greater than 0.50, which indicates that the results of the convergent validity fit the standards.

### 3.4. Reliability Analysis Results

Cronbach's alpha coefficients for the three dimensions were 0.920, 0.877, and 0.871, respectively, and Cronbach's alpha coefficient for the total scale was 0.939. The Spearman–Brown coefficients for the three dimensions were 0.922, 0.872, and 0.872, respectively, and the Spearman–Brown coefficient for the total scale was 0.908 (Table 5).

## 4. Discussion

This study aimed to develop and test the psychometric properties of a scale for organizational support needs for nurses returning to work after childbirth. A study in the United States found that perceived breastfeeding support was positive among nurses returning to work after childbirth, and that adequate rest time was positively associated with breastfeeding duration [50]. An in-depth exploration of the breastfeeding experiences of nurses returning to work revealed that occupational hazards were one of the nurses' concerns, fearing that infectious diseases could infect their babies through breast milk. There was also a need for a breastfeeding room and support from colleagues while expressing breast milk at work [51]. These findings suggest that organizational support is essential for nurses to achieve their breastfeeding goals. Nurses felt that there was a lack of communication with the organization regarding returning to work, and therefore, did not feel supported by the organization [52]. Two instruments were developed for nurses returning after childbirth: the Work Pressure Scale for Postpartum Nurses Who Returned to Work [53], which measures the stress of nurses returning to work after childbirth, and the Postpartum Return Adaptation Questionnaire for Nurses [28], which assesses nurses' adaptation to returning to work after childbirth. However, there is a lack of scales to assess the organizational support needs of nurses returning to work after childbirth. Therefore, further studies are warranted. The development of a scientific management plan by nursing managers requires a proper assessment of the organizational support needs of nurses returning to work after childbirth.

The first step in scale development is item development. In this study, items were extracted from the results of interviews with participants through qualitative descriptive research and supplemented by a literature review. In the qualitative study phase, the interview outline served as a framework to conduct semistructured in-depth interviews using four factors that influenced perceptions of organizational support as the orientation for questions. This approach ensured that the interviews remained focused on the topic and were comprehensive. Regarding the issue of organizational support for nurses returning to work after childbirth, in the absence of quantitative studies, qualitative descriptive research was well-suited to gain insight into the issue and extract items [54]. The literature review helped researchers understand the organizational support needs of nurses in other geographic areas, supplementing the items. Initially, the dimensions were named according to the practical meaning of the items, drawing on OST to make the names of the dimensions more evidence-based.

In this study, 15 experts from nine different provinces and cities across the country were invited to conduct two rounds of Delphi expert consultations. The Delphi method is particularly important in scale development. The Delphi expert consultation enables a consensus to be reached on research issues through expert opinions [31]. Implementing the Delphi expert consultation by mail allows a panel of experts from different geographic locations to answer research questions, breaking through geographic limitations and making the results geographically representative [55]. Strict anonymity was guaranteed during the two rounds of expert consultation to ensure the credibility and reliability of the results. In addition, we invited another six experts to assess the correlations between the items and the scales and to analyze content validity. The I-CVI of the scale ranged from 0.83 to 1.00, and the S-CVI/Ave was 0.92. When the number of experts is between six and eight, I-CVI should be greater than 0.83 [34]. The S-CVI/Ave ratio was > 0.90 [35]. Therefore, this scale had good content validity.

This study utilized EFA to analyze the internal structure of latent variables and assessed the construct validity of the scale, which is the proper use of EFA [56]. After two EFAs, one item each with factor loadings less than 0.40 was deleted. According to the Kaiser method [57], the third EFA extracted three factors with eigenvalues greater than one and a cumulative variance contribution rate of 72.912%. The scale structure was stabilized at 14 items and 3 dimensions, and the attribution of the items was the same as in the Delphi expert consultation phase, so the naming of the dimensions was not modified. After EFA, the internal consistency of the scales for the remaining items was assessed. Cronbach's alpha coefficient for the total scale was 0.939, and those for the three dimensions were 0.920, 0.877, and 0.871. The Spearman–Brown coefficient for the total scale was 0.908, and the Spearman–Brown coefficients for the three dimensions were 0.922, 0.872, and 0.872. Cronbach's alpha coefficient and the Spearman–Brown coefficient range between 0 and 1, with a larger value indicating higher internal consistency [58]. Therefore, the scale had good internal consistency. Finally, the relationships between the items and dimensions of the scales were examined through SEM with CFA. It was found that the structure of the scale and the model fit well. Therefore, the scale has good structural validity.

## 5. Conclusions

The scale of organizational support needs for nurses returning to work after childbirth was developed using scientific methods and has good reliability and validity. The scale contains three dimensions (manager support, work condition support, and colleague support) and 14 items that provide a tool for nursing managers to evaluate the organizational support needs of nurses returning to work after childbirth.

### 5.1. Limitations

This scale has not been applied and could be used to investigate the current situation of organizational support demands of nurses returning to work after childbirth and analyze its influencing factors in the future.

## Figures and Tables

**Table 1:** Demographic characteristics (*n* = 414).

Variables	*n*	(%)
*Age*		
20–30	161	38.89
31–39	241	58.21
> 39	12	2.90

*Nation*		
Han Chinese	358	86.47
Ethnic minorities	56	13.53

*Working years*		
0–9	240	57.97
> 9	174	42.03

*Professional title*		
Junior title	286	69.08
Intermediate title	121	29.23
Associate senior title	7	1.69

*Education*		
College	77	18.60
Bachelor's degree	326	78.74
Master's degree	11	2.66

*Number of births*		
First birth	353	85.27
Second birth	61	14.73

**Table 2:** Results of item analysis.

Item	Critical ratio decision value		Item-total correlation coefficient		Cronbach's *α* if item deleted
	CR	*p*	*r*	*p*	
*Manager support*					
1. I need to be assigned to a position with a light workload.	14.988	0.001	0.731	< 0.001	0.937
2. I need a flexible schedule for lactation leave.	15.548	0.001	0.757	< 0.001	0.937
3. I need the manager to grant my request for leave at her/his discretion.	1.611	**0.110**			
4. I need the manager to understand that I express breast milk during work.	16.811	0.001	0.747	< 0.001	0.937
5. I need a good working atmosphere.	1.030	**0.305**			
6. I need to leave work without delay.	18.112	0.001	0.778	< 0.001	0.936
7. I need to postpone the recovery time for night shift scheduling.	20.380	0.001	0.767	< 0.001	0.936
8. I need to extend my nursing leave (1 hour a day).	15.761	0.001	0.754	< 0.001	0.937
9. I need supportive psychological help from the manager (listening, explaining, advising, etc.).	−1.462	**0.147**			
10. I hope the manager supervises my work more.	1.466	**0.146**			

*Work condition support*					
11. Provide me with child care day care (child care services).	0.573	**0.568**			
12. Provide me with a quiet and secluded space for breastfeeding.	15.562	0.001	0.720	< 0.001	0.938
13. Provide me with milk storage equipment.	1.504	**0.136**			
14. Provide me with a variety of working meals.	−1.665	**0.099**			
15. Provide me with protective conditions that reduce bacteria and viruses.	13.009	0.001	0.672	< 0.001	0.939
16. Provide me with protective conditions that reduce radiation.	20.103	0.001	0.782	< 0.001	0.937
17. I need to train and learn after returning to work.	14.670	0.001	0.682	< 0.001	0.939
18. I needed an adjustment period after returning to work.	12.021	0.001	0.600	< 0.001	0.940
19. I need to choose my department when returning to work.	11.834	0.001	0.595	< 0.001	0.940

*Colleague support*					
20. I need my colleagues to understand that I express breast milk during work.	14.244	0.001	0.654	< 0.001	0.939
21. I hope my colleagues to help me with my work while I am expressing breast milk.	13.630	0.001	0.678	< 0.001	0.939
22. I hope my colleagues share their parenting experiences.	14.604	0.001	0.686	< 0.001	0.938
23. I hope my colleagues to switch shifts with me when necessary.	11.590	0.001	0.594	< 0.001	0.940
24. I need supportive psychological help from colleagues (listening, explaining, advising, etc.).	−0.259	**0.796**			

*Note:* The bolded values (*p* > 0.05) indicate nonsignificant results, suggesting no statistically significant differences were found in this analysis.

**Table 3:** Results of the third EFA.

Item	Factor 1	Factor 2	Factor 3
6. I need to leave work without delay.	0.820		
4. I need the manager to understand that I express breast milk during work.	0.817		
7. I need to postpone the recovery time for night shift scheduling.	0.786		
2. I need a flexible schedule for lactation leave.	0.760		
1. I need to be assigned to a position with a light workload.	0.725		
8. I need to extend my nursing leave (1 hour a day).	0.719		
22. I hope my colleagues share their parenting experiences.		0.873	
21. I hope my colleagues to help me with my work while I am expressing breast milk.		0.834	
20. I need my colleagues to understand that I express breast milk during work.		0.783	
23. I hope my colleagues to switch shifts with me when necessary.		0.612	
16. Provide me with protective conditions that reduce radiation.			0.874
15. Provide me with protective conditions that reduce bacteria and viruses.			0.769
12. Provide me with a quiet and secluded space for breastfeeding.			0.761
17. I need to train and learn after returning to work.			0.453
The eigenvalues of the factors	7.812	1.326	1.070
The variances explained by the eigenvalues (%)	55.797	9.474	7.641
The cumulative variance values explained by the eigenvalues (%)		72.912	

**Table 4:** Results of convergent validity.

Dimensions	Item	Standardized factor loading	AVE value	CR value
Manager support	Item 1	0.7690	0.6226	0.9081
	Item 2	0.7639		
	Item 4	0.8037		
	Item 6	0.8146		
	Item 7	0.8193		
	Item 8	0.7614		

Work condition support	Item 12	0.8150	0.6139	0.8616
	Item 15	0.8094		
	Item 16	0.8919		
	Item 17	0.5839		

Colleague support	Item 20	0.7466	0.5721	0.8413
	Item 21	0.8275		
	Item 22	0.7980		
	Item 23	0.6399		

**Table 5:** Results of reliability analysis.

Dimensions	Cronbach's *α* coefficient	Spearman–Brown coefficient
Manager support	0.920	0.922
Work condition support	0.877	0.872
Colleague support	0.871	0.872
Total scale	0.939	0.908

## Data Availability

The data that support the findings of this study are available from the corresponding author upon reasonable request.
